# Harmful mutation load in the mitochondrial genomes of cattle breeds

**DOI:** 10.1186/s13104-021-05664-y

**Published:** 2021-06-27

**Authors:** Sankar Subramanian

**Affiliations:** GeneCology Research Centre, School of Science, Technology and Engineering, The University of the Sunshine Coast, 1 Moreton Parade, Petrie, Moreton Bay, QLD 4502 Australia

**Keywords:** Mutation load, Deleterious mutations, Cattle breeds, Inbreeding

## Abstract

**Objective:**

Domestication of wild animals results in a reduction in the effective population size, and this could affect the deleterious mutation load of domesticated breeds. Furthermore, artificial selection will also contribute to the accumulation of deleterious mutations due to the increased rate of inbreeding among these animals. The process of domestication, founder population size, and artificial selection differ between cattle breeds, which could lead to a variation in their deleterious mutation loads. We investigated this using mitochondrial genome data from 364 animals belonging to 18 cattle breeds of the world.

**Results:**

Our analysis revealed more than a fivefold difference in the deleterious mutation load among cattle breeds. We also observed a negative correlation between the breed age and the proportion of deleterious amino acid-changing polymorphisms. This suggests a proportionally higher deleterious SNPs in young breeds compared to older breeds. Our results highlight the magnitude of difference in the deleterious mutations present in the mitochondrial genomes of various breeds. The results of this study could be useful in predicting the rate of incidence of genetic diseases in different breeds.

**Supplementary Information:**

The online version contains supplementary material available at 10.1186/s13104-021-05664-y.

## Introduction

Domestication of wild animals results in a drastic reduction of effective population size as only a subsample of the wild population is used. Artificial selection further reduces the population size due to inbreeding within domesticated animals, which occurs during breed formation [1]. The reduction in the population size leads to the accumulation of deleterious variants as selection is not efficient in removing them owing to genetic drift in small populations [2]. Previous studies showed empirical evidence for these predictions by using the ratio (*ω*) of the diversity of amino acid replacement (nonsynonymous) polymorphisms and the diversity of silent (synonymous) polymorphisms as the measure of deleterious mutational load [3–6]. Since most of the replacement polymorphisms affect protein structure and function, they are harmful to the organism. In contrast, silent polymorphisms are neutral—have no effect on proteins. Hence higher *ω* suggests elevated deleterious mutation load. Earlier studies revealed a much higher *ω* for domesticated pig, dog, rabbit, silkworm, rice and sunflower compared to their wild relatives [3–5, 7, 8]. Furthermore, the *ω* estimated for different breeds of dogs were found to correlate with their silent diversity [5] and similar results were reported for the breeds of pig, rabbit and chicken [4]. Since the process of artificial selection, level of inbreeding and the number of founder animals used vary between cattle breeds, the harmful mutational load is also expected to differ among breeds. Furthermore, the deleterious mutations were found to correlate negatively with the age of human haplogroups [9]. Young haplogroups contained more deleterious mutations than old ones.

The present study is focused on investigating this by estimating the deleterious mutational load of the mitochondrial genomes of 18 cattle breeds and comparing them with the age of the breeds.

## Main text

### Methods

#### Mitogenome data

A total of 578 mitochondrial genomes of *Bos taurus* were downloaded from GenBank (https://www.ncbi.nlm.nih.gov/genbank/) by searching using the keywords *Bos taurus*, *mitochondrion*, and *complete genome*. The records without specific breed information were excluded. Furthermore, only the breeds that have mitochondrial genomes from more than four individuals were included. To reduce estimation errors, breeds having at least one synonymous and nonsynonymous variant or SNP were included. This resulted in 364 mitogenomes belonging to 18 breeds. The accession number and locations are given in Additional file 1: Table S1. The haplogroups of breeds and their age (of their common ancestor) are given in Additional file 1: Table S2. Using in-house Perl scripts, 13 mitochondrial protein-coding genes were extracted from the GenBank records using the annotations. Sequences from each protein-coding gene from 364 cattle were aligned using the program *mafft* [10]—online version (https://mafft.cbrc.jp/alignment/server/). After alignment, all 13 protein-coding genes (CDS) were to form a super alignment containing 11,229 base pairs from 364 animals.

#### Data analysis

The CDS alignment was then used to estimate nonsynonymous (*π*_N_) and synonymous (*π*_S_) diversities using the Pamilo-Bianchi-Li method [11, 12] employed in the software program MEGA—10.2.2 [13]. The ratio of these measures (*ω*_*P*_ = *π*_N_/*π*_S_) for each breed was estimated. Furthermore, the ratio (*ω*_*S*_) of interspecies synonymous (*d*_S_) and nonsynonymous (*d*_N_) divergence between cow and bison mitochondrial protein-coding genes was also computed (*ω*_*S*_ = *d*_N_/*d*_S_). Using the population and species level estimates of the ratios, it is possible to calculate the proportion of deleterious mutations (*δ*) segregating in a population using the following formula [14, 15]:

$$\delta =\frac{{{\omega }_{P}-\omega }_{S}}{{\omega }_{P}}$$

To estimate the age of the breed populations, the program *BP&P* was used [16]. To convert the breed coalescence distance into time, we used the mitochondrial mutation rate of 2.043 × 10^–8^ substitutions per site [17, 18]. The bootstrap resampling procedure was utilized (using 100 replications) to calculate the variance for diversity and divergence estimates. Pearson correlation coefficient was used to measure the strength and significance of correlations. However, using the nonparametric Spearman rank correlation also showed similar strength of correlations.

### Results and discussion

**Fig. 1:**
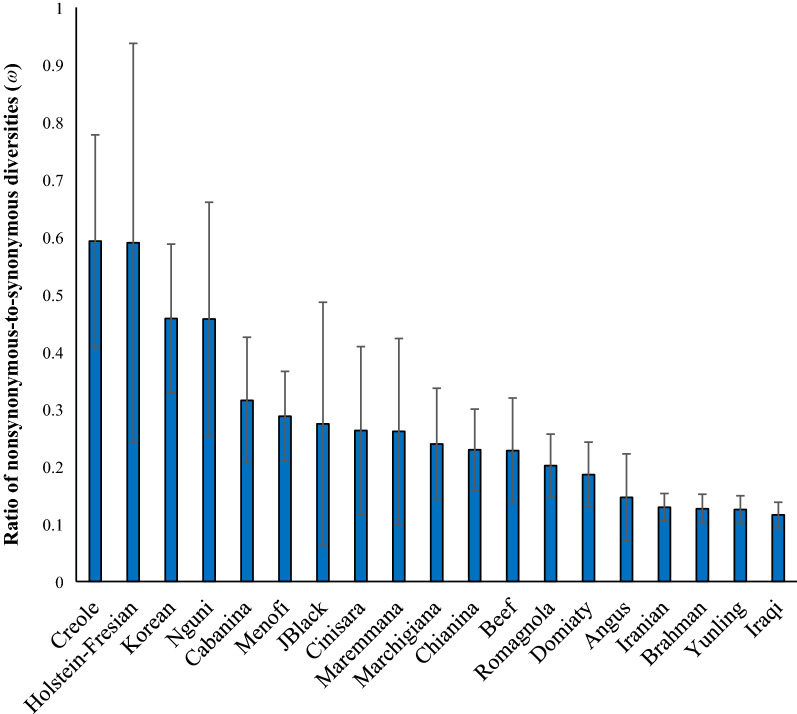
The ratio of replacement (nonsynonymous) diversity to silent (synonymous) diversity (*ω*_*P*_) was estimated using 13 mitochondrial protein-coding genes of 364 cattle belonging to 18 breeds. The error bars denote the standard error of the mean that were estimated using the bootstrap resampling procedure

The complete mitochondrial genome sequences of 364 individuals belonging to 18 cattle breeds were obtained from GenBank (Additional file 1: Table S1). The nonsynonymous (*π*_N_) and synonymous (*π*_S_) diversities were estimated for the concatenated alignment of 13 mitochondrial protein-coding genes, and their ratio (*ω*_*P*_ = *π*_N_/*π*_S_) was calculated for each breed population. As shown in Fig. 1, *ω*_*P*_ varies more than fivefold (5.1) among cattle breeds, with Creole having the highest *ω*_*P*_ of 0.59 and the lowest was observed for Iraqi (0.116). This suggests much higher harmful mutations are segregating in the breed Creole compared to their Iraqi counterparts.

To accurately quantify the harmful mutations, we calculated the proportion of harmful mutations (*δ*) segregating in each breed using the equation (see “Methods”) developed by a previous study [14, 15]. This was accomplished by estimating the ratio of the interspecies nonsynonymous-to-synonymous divergence or substitutions (*ω*_*S*_ = *d*_N_/*d*_S_) by comparing the mitochondrial protein-coding genes of cow and bison. While *ω*_*S*_ indicates the proportion of nonsynonymous mutations fixed in the cow lineage, *ω*_*P*_ denotes the proportion of nonsynonymous mutations or SNPs segregating in the cattle populations. Population genetic theories predict that neutral (mutations with no harmful effects) mutations are fixed in a species. Therefore, the value of *ω*_*S*_ suggests the proportion of neutral nonsynonymous mutations fixed in cattle. If *ω*_*P*_ = *ω*_*S*_, then the nonsynonymous SNPs segregating in cow breeds are effectively neutral. However, *ω*_*P*_ > *ω*_*S*_ suggests an excess of nonsynonymous SNPs segregating in cattle breed populations. This excess SNPs are potentially deleterious, and they will be removed from the population over time. The deleterious excess fraction could be calculated using the equation in “Methods” section (for mathematical derivation—see Subramanian [6]).

**Fig. 2:**
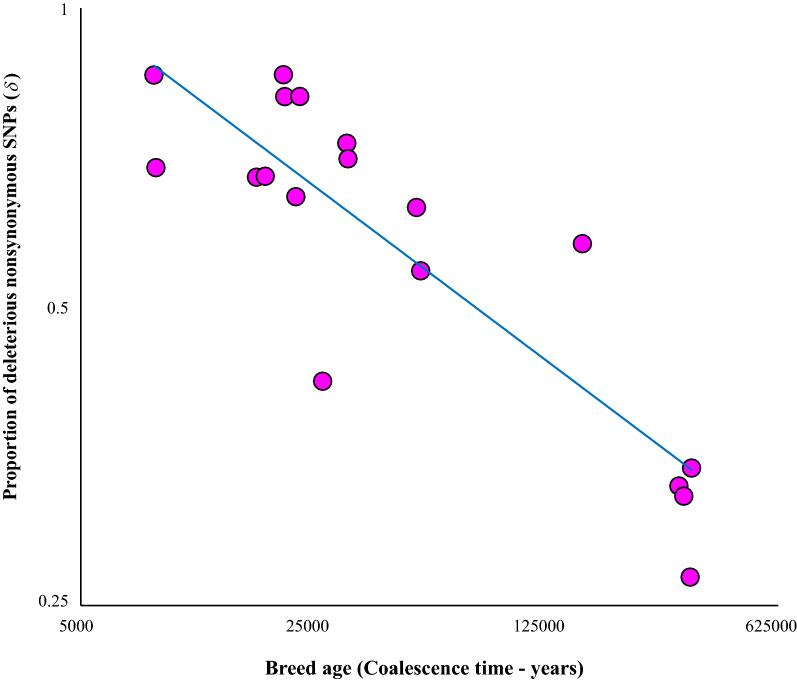
Relationship breed age and the fraction of deleterious nonsynonymous SNPs in 18 cattle breeds. X and Y axes show log-transformed values. The correlation was highly significant (Pearson *r* = − 0.86, *P* = 0.000005). The best fitting regression line is shown

To further elucidate the potential reason for the observed variation in the deleterious nonsynonymous SNPs among breeds, the age of the breeds was estimated using Bayesian population coalescence methods [16]. The age estimates of breeds obtained in this study are very similar to previous estimates [17, 18]. The breed ages were then plotted against the proportion of deleterious SNPs (*δ*) and a highly significant negative correlation was observed (Pearson *r* = − 0.86, *P* = 0.000005) (Fig. 2). This suggests that young breeds have more deleterious mutations than older breeds. For instance, the age of the common ancestor of the Creole breed is 8.3 Kyrs, and 86% of the nonsynonymous SNPs segregating in this are deleterious in nature. On the contrary, only 27% of the nonsynonymous SNPs are deleterious in the Iraqi breed, which is 340 Kyr old.

A deeper look at the haplogroups of the mitogenomes revealed that young breeds belong to only a single haplogroup, whereas older breeds belong to broader haplogroups. For instance, the age of Creole and Japanese Black are around 8.3 Kyrs, and they exclusively belong to T1 and T3 haplogroups, respectively (Additional file 1: Table S2). However, those of the intermediate ages (16–32 Kyrs) belong to more than one haplogroup (T1, T2, T3 etc.) but within the T haplogroup and their common ancestral node was T [18]. Most of the Italian breeds, Holstein–Friesian and Korean, are in this category. The breeds such as Domiaty and Chianina are 51–52 Kyrs old and belong to T and Q haplogroups and their common ancestral node was QT [18]. Whereas the much older (160 Kyr) Romagnola breed consists of T, Q and R haplogroups, which have the common ancestor, REPQT [17, 18]. Finally, the oldest (> 300 Kyrs) Brahman, Yunling, Irani, Iraqi are cross between taurine and indicine cattle. Hence, they have individuals belonging to haplogroups I1 and I2 (indicine) as well as T1–T5 (taurine), and their common ancestral node is referred to REPQTI [17, 18]. Figure 2 shows a negative correlation between the fraction of deleterious SNPs and the breadth of haplogroups. A fraction of deleterious SNPs was much higher in breeds belonging to a single haplogroup than those belonging to multiple and diversified haplogroups.

It is well known that deleterious mutations segregate in young populations, and over time they are removed by natural selection. This has been well demonstrated in human populations, and the young European haplogroups such as H and T have more deleterious mutations than the older African haplogroups such as L0 and L1 [9]. Our results highlight the magnitude of difference in the deleterious mutations present in the mitochondrial genomes of various breeds. Similar results were observed for the nuclear genes of pig, rabbit, chicken and dog breeds [4, 5]. In this study, the deleterious mutation loads of various breeds were determined based on nonsynonymous polymorphisms. However, a similar pattern is expected for the mutations causing major genetic diseases in cattle.

## Conclusions

The present study revealed that the deleterious mutation load significantly varies between different cattle breeds. Hence the results of this study could be useful in predicting the rate of incidence of mitochondrial genetic diseases in various breeds.

## Limitations

This study is based on mitochondrial genomes that are uniparentally inherited. Furthermore, recombination is not well documented in mitogenomes. Therefore, it is unclear whether a similar pattern of mutation load is expected in nuclear genomes. Hence, further studies based on large number of nuclear genomes is required to investigate this.

## Supplementary Information

**Additional file 1: Table S1.** List of Genbank accession numbers and breed information. **Table S2.** Haplogroups and age of breeds

## Data Availability

The data used in this work can be obtained from GenBank using the list of accession numbers in Additional file 1: Table S1. The Perl scripts used in this study will be available upon request.

## Abbreviation

MEGA: Molecular Evolutionary Genetic Analysis
