# Supplementary material for: Harmful mutation load in the mitochondrial genomes of cattle breeds
Source: BMC Res Notes. 2021 Jun 27;14:241. doi: 10.1186/s13104-021-05664-y (PMC8237412; doi:10.1186/s13104-021-05664-y)
Supplement: Supplementary file 1 — Additional file 1: Table S1. List of Genbank accession numbers and breed information. Table S2. Haplogroups and age of breeds [file 13104_2021_5664_MOESM1_ESM.pdf]

Harmful mutational load in the mitogenomes of cattle breeds

Sankar Subramanian

GeneCology Research Centre, School of Science and Engineering, The University of the Sunshine Coast, Moreton Bay, QLD 4502, Australia

Supporting Information

Table S1. List of Mitochondrial genomes used in the study

Accession	Breed	Subspecies	Origin
AY676873	Angus	Taurine	USA
AY676872	Angus	Taurine	USA
AY676871	Angus	Taurine	USA
AY676870	Angus	Taurine	USA
AY676869	Angus	Taurine	USA
AY676868	Angus	Taurine	USA
AY676867	Angus	Taurine	USA
AY676866	Angus	Taurine	USA
AY676865	Angus	Taurine	USA
AY676864	Angus	Taurine	USA
AY676863	Angus	Taurine	USA
AY676862	Angus	Taurine	USA
AY676859	Angus	Taurine	USA
AY676858	Angus	Taurine	USA
AY676857	Angus	Taurine	USA
MN200889	Brahman	Taurine-Indicine	USA
MN200875	Brahman	Taurine-Indicine	USA
MN200874	Brahman	Taurine-Indicine	USA
MN200838	Brahman	Taurine-Indicine	USA
MN200836	Brahman	Taurine-Indicine	USA
MN200834	Brahman	Taurine-Indicine	USA
MN200833	Brahman	Taurine-Indicine	USA
MN200832	Brahman	Taurine-Indicine	USA
MN200831	Brahman	Taurine-Indicine	USA
MN200830	Brahman	Taurine-Indicine	USA
MN200829	Brahman	Taurine-Indicine	USA
MN200828	Brahman	Taurine-Indicine	USA
MN200827	Brahman	Taurine-Indicine	USA
MN200826	Brahman	Taurine-Indicine	USA
MN200825	Brahman	Taurine-Indicine	USA
MN200824	Brahman	Taurine-Indicine	USA
MN200823	Brahman	Taurine-Indicine	USA
MN200821	Brahman	Taurine-Indicine	USA
MN200820	Brahman	Taurine-Indicine	USA
MN200819	Brahman	Taurine-Indicine	USA
MN200818	Brahman	Taurine-Indicine	USA
MN200817	Brahman	Taurine-Indicine	USA
MN200815	Brahman	Taurine-Indicine	USA
MN200814	Brahman	Taurine-Indicine	USA
MN200813	Brahman	Taurine-Indicine	USA
MN200812	Brahman	Taurine-Indicine	USA
MN200810	Brahman	Taurine-Indicine	USA
MN200809	Brahman	Taurine-Indicine	USA
MN200791	Brahman	Taurine-Indicine	USA
MN200786	Brahman	Taurine-Indicine	USA
MN200785	Brahman	Taurine-Indicine	USA
EU177840	Cabannina	Taurine	Italy
EU177850	Cabannina	Taurine	Italy
EU177851	Cabannina	Taurine	Italy
EU177866	Cabannina	Taurine	Italy
EU177867	Cabannina	Taurine	Italy

HQ184038	Cabannina	Taurine	Italy
FJ971081	Chianina	Taurine	Italy
JN817350	Chianina	Taurine	Italy
JN817316	Chianina	Taurine	Italy
JN817315	Chianina	Taurine	Italy
JN817314	Chianina	Taurine	Italy
JN817313	Chianina	Taurine	Italy
EU177855	Chianina	Taurine	Italy
EU177854	Chianina	Taurine	Italy
EU177853	Chianina	Taurine	Italy
EU177846	Chianina	Taurine	Italy
EU177845	Chianina	Taurine	Italy
EU177841	Chianina	Taurine	Italy
EU177828	Chianina	Taurine	Italy
EU177825	Chianina	Taurine	Italy
EU177822	Chianina	Taurine	Italy
EU177820	Chianina	Taurine	Italy
EU177819	Chianina	Taurine	Italy
EU177818	Chianina	Taurine	Italy
EU177816	Chianina	Taurine	Italy
HQ184039	Chianina	Taurine	Italy
HQ184032	Chianina	Taurine	Italy
HQ184031	Chianina	Taurine	Italy
HQ184030	Chianina	Taurine	Italy
FJ971086	Cinisara	Taurine	Italy
FJ971085	Cinisara	Taurine	Italy
JN817320	Cinisara	Taurine	Italy
JN817319	Cinisara	Taurine	Italy
JN817318	Cinisara	Taurine	Italy
JN817317	Cinisara	Taurine	Italy
EU177842	Cinisara	Taurine	Italy
EU177823	Cinisara	Taurine	Italy
JN817307	Creole	Taurine	Mexico/Paraguay
JN817308	Creole	Taurine	Mexico/Paraguay
JN817309	Creole	Taurine	Mexico/Paraguay
JN817310	Creole	Taurine	Mexico/Paraguay
JN817311	Creole	Taurine	Mexico/Paraguay
KT184472	Domiaty	Taurine	Egypt
KT184471	Domiaty	Taurine	Egypt
KT184470	Domiaty	Taurine	Egypt
KT184469	Domiaty	Taurine	Egypt
KT184463	Domiaty	Taurine	Egypt
KT184462	Domiaty	Taurine	Egypt
KT184460	Domiaty	Taurine	Egypt
KT184457	Domiaty	Taurine	Egypt
KT184456	Domiaty	Taurine	Egypt
KT184452	Domiaty	Taurine	Egypt
JN817324	Domiaty	Taurine	Egypt
JN817323	Domiaty	Taurine	Egypt
JN817322	Domiaty	Taurine	Egypt
JN817321	Domiaty	Taurine	Egypt
DQ124418	Holstein-Friesian	Taurine	Northwest Europe
DQ124417	Holstein-Friesian	Taurine	Northwest Europe
DQ124416	Holstein-Friesian	Taurine	Northwest Europe

DQ124415	Holstein-Friesian	Taurine	Northwest Europe
DQ124414	Holstein-Friesian	Taurine	Northwest Europe
DQ124413	Holstein-Friesian	Taurine	Northwest Europe
DQ124412	Holstein-Friesian	Taurine	Northwest Europe
DQ124411	Holstein-Friesian	Taurine	Northwest Europe
DQ124410	Holstein-Friesian	Taurine	Northwest Europe
DQ124409	Holstein-Friesian	Taurine	Northwest Europe
DQ124408	Holstein-Friesian	Taurine	Northwest Europe
DQ124407	Holstein-Friesian	Taurine	Northwest Europe
DQ124406	Holstein-Friesian	Taurine	Northwest Europe
DQ124405	Holstein-Friesian	Taurine	Northwest Europe
DQ124404	Holstein-Friesian	Taurine	Northwest Europe
DQ124403	Holstein-Friesian	Taurine	Northwest Europe
EU177870	Iranian	Taurine-Indicine	Iranian
EU177860	Iranian	Taurine-Indicine	Iranian
EU177859	Iranian	Taurine-Indicine	Iranian
EU177858	Iranian	Taurine-Indicine	Iranian
EU177857	Iranian	Taurine-Indicine	Iranian
EU177839	Iranian	Taurine-Indicine	Iranian
EU177838	Iranian	Taurine-Indicine	Iranian
EU177869	Iraqi	Taurine-Indicine	Iraqi
EU177868	Iraqi	Taurine-Indicine	Iraqi
EU177865	Iraqi	Taurine-Indicine	Iraqi
EU177864	Iraqi	Taurine-Indicine	Iraqi
EU177856	Iraqi	Taurine-Indicine	Iraqi
EU177848	Iraqi	Taurine-Indicine	Iraqi
EU177837	Iraqi	Taurine-Indicine	Iraqi
EU177836	Iraqi	Taurine-Indicine	Iraqi
EU177835	Iraqi	Taurine-Indicine	Iraqi
AB074968	Japanese Black	Taurine	Japan
AB074967	Japanese Black	Taurine	Japan
AB074966	Japanese Black	Taurine	Japan
AB074965	Japanese Black	Taurine	Japan
AB074964	Japanese Black	Taurine	Japan
AB074963	Japanese Black	Taurine	Japan
AB074962	Japanese Black	Taurine	Japan
DQ124386	Korean cattle	Taurine	Korea
DQ124385	Korean cattle	Taurine	Korea
DQ124384	Korean cattle	Taurine	Korea
DQ124383	Korean cattle	Taurine	Korea
DQ124382	Korean cattle	Taurine	Korea
DQ124381	Korean cattle	Taurine	Korea
DQ124380	Korean cattle	Taurine	Korea
DQ124379	Korean cattle	Taurine	Korea
DQ124378	Korean cattle	Taurine	Korea
DQ124377	Korean cattle	Taurine	Korea
DQ124376	Korean cattle	Taurine	Korea
DQ124375	Korean cattle	Taurine	Korea
DQ124374	Korean cattle	Taurine	Korea
DQ124373	Korean cattle	Taurine	Korea
DQ124372	Korean cattle	Taurine	Korea
DQ124371	Korean cattle	Taurine	Korea
JN817351	Marchigiana	Taurine	Italy
JN817339	Marchigiana	Taurine	Italy

JN817338	Marchigiana	Taurine	Italy
JN817337	Marchigiana	Taurine	Italy
JN817336	Marchigiana	Taurine	Italy
JN817335	Marchigiana	Taurine	Italy
JN817334	Marchigiana	Taurine	Italy
HQ184045	Marchigiana	Taurine	Italy
EU177824	Maremmana	Taurine	Italy
EU177829	Maremmana	Taurine	Italy
EU177844	Maremmana	Taurine	Italy
JN817332	Maremmana	Taurine	Italy
JN817333	Maremmana	Taurine	Italy
KT184468	Menofi	Taurine	Egypt
KT184467	Menofi	Taurine	Egypt
KT184466	Menofi	Taurine	Egypt
KT184465	Menofi	Taurine	Egypt
KT184464	Menofi	Taurine	Egypt
KT184461	Menofi	Taurine	Egypt
KT184459	Menofi	Taurine	Egypt
KT184458	Menofi	Taurine	Egypt
KT184455	Menofi	Taurine	Egypt
KT184454	Menofi	Taurine	Egypt
KT184453	Menofi	Taurine	Egypt
KT184451	Menofi	Taurine	Egypt
JN817329	Menofi	Taurine	Egypt
JN817328	Menofi	Taurine	Egypt
JN817327	Menofi	Taurine	Egypt
JN817326	Menofi	Taurine	Egypt
JN817325	Menofi	Taurine	Egypt
KF163094	Nguni	Taurine	Africa
KF163093	Nguni	Taurine	Africa
KF163092	Nguni	Taurine	Africa
KF163091	Nguni	Taurine	Africa
KF163090	Nguni	Taurine	Africa
KF163089	Nguni	Taurine	Africa
KF163088	Nguni	Taurine	Africa
KF163087	Nguni	Taurine	Africa
KF163086	Nguni	Taurine	Africa
KF163085	Nguni	Taurine	Africa
KF163084	Nguni	Taurine	Africa
KF163083	Nguni	Taurine	Africa
KF163082	Nguni	Taurine	Africa
KF163081	Nguni	Taurine	Africa
KF163080	Nguni	Taurine	Africa
KF163079	Nguni	Taurine	Africa
KF163078	Nguni	Taurine	Africa
KF163077	Nguni	Taurine	Africa
KF163076	Nguni	Taurine	Africa
KF163075	Nguni	Taurine	Africa
KF163074	Nguni	Taurine	Africa
KF163073	Nguni	Taurine	Africa
KF163072	Nguni	Taurine	Africa
KF163071	Nguni	Taurine	Africa
KF163070	Nguni	Taurine	Africa
KF163069	Nguni	Taurine	Africa

KF163068	Nguni	Taurine	Africa
KF163067	Nguni	Taurine	Africa
KF163066	Nguni	Taurine	Africa
KF163065	Nguni	Taurine	Africa
KF163064	Nguni	Taurine	Africa
KF163063	Nguni	Taurine	Africa
KF163062	Nguni	Taurine	Africa
KF163061	Nguni	Taurine	Africa
FJ971087	Romagnola	Taurine	Italy
FJ971083	Romagnola	Taurine	Italy
FJ971080	Romagnola	Taurine	Italy
JN817347	Romagnola	Taurine	Italy
JN817346	Romagnola	Taurine	Italy
JN817345	Romagnola	Taurine	Italy
HQ184044	Romagnola	Taurine	Italy
HQ184043	Romagnola	Taurine	Italy
HQ184042	Romagnola	Taurine	Italy
HQ184041	Romagnola	Taurine	Italy
HQ184040	Romagnola	Taurine	Italy
HQ184035	Romagnola	Taurine	Italy
HQ184034	Romagnola	Taurine	Italy
HQ184033	Romagnola	Taurine	Italy
MN200938	Yunling	Taurine-Indicine	China
MN200937	Yunling	Taurine-Indicine	China
MN200936	Yunling	Taurine-Indicine	China
MN200935	Yunling	Taurine-Indicine	China
MN200934	Yunling	Taurine-Indicine	China
MN200933	Yunling	Taurine-Indicine	China
MN200932	Yunling	Taurine-Indicine	China
MN200931	Yunling	Taurine-Indicine	China
MN200930	Yunling	Taurine-Indicine	China
MN200929	Yunling	Taurine-Indicine	China
MN200928	Yunling	Taurine-Indicine	China
MN200927	Yunling	Taurine-Indicine	China
MN200926	Yunling	Taurine-Indicine	China
MN200925	Yunling	Taurine-Indicine	China
MN200924	Yunling	Taurine-Indicine	China
MN200923	Yunling	Taurine-Indicine	China
MN200922	Yunling	Taurine-Indicine	China
MN200921	Yunling	Taurine-Indicine	China
MN200920	Yunling	Taurine-Indicine	China
MN200919	Yunling	Taurine-Indicine	China
MN200918	Yunling	Taurine-Indicine	China
MN200917	Yunling	Taurine-Indicine	China
MN200916	Yunling	Taurine-Indicine	China
MN200915	Yunling	Taurine-Indicine	China
MN200914	Yunling	Taurine-Indicine	China
MN200913	Yunling	Taurine-Indicine	China
MN200912	Yunling	Taurine-Indicine	China
MN200911	Yunling	Taurine-Indicine	China
MN200910	Yunling	Taurine-Indicine	China
MN200909	Yunling	Taurine-Indicine	China
MN200908	Yunling	Taurine-Indicine	China
MN200907	Yunling	Taurine-Indicine	China

MN200906	Yunling	Taurine-Indicine	China
MN200905	Yunling	Taurine-Indicine	China
MN200904	Yunling	Taurine-Indicine	China
MN200903	Yunling	Taurine-Indicine	China
MN200902	Yunling	Taurine-Indicine	China
MN200901	Yunling	Taurine-Indicine	China
MN200900	Yunling	Taurine-Indicine	China
MN200899	Yunling	Taurine-Indicine	China
MN200898	Yunling	Taurine-Indicine	China
MN200897	Yunling	Taurine-Indicine	China
MN200896	Yunling	Taurine-Indicine	China
MN200895	Yunling	Taurine-Indicine	China
MN200894	Yunling	Taurine-Indicine	China
MN200893	Yunling	Taurine-Indicine	China
MN200892	Yunling	Taurine-Indicine	China
MN200891	Yunling	Taurine-Indicine	China
MN200890	Yunling	Taurine-Indicine	China
MN200888	Yunling	Taurine-Indicine	China
MN200887	Yunling	Taurine-Indicine	China
MN200886	Yunling	Taurine-Indicine	China
MN200885	Yunling	Taurine-Indicine	China
MN200884	Yunling	Taurine-Indicine	China
MN200883	Yunling	Taurine-Indicine	China
MN200882	Yunling	Taurine-Indicine	China
MN200881	Yunling	Taurine-Indicine	China
MN200880	Yunling	Taurine-Indicine	China
MN200879	Yunling	Taurine-Indicine	China
MN200878	Yunling	Taurine-Indicine	China
MN200877	Yunling	Taurine-Indicine	China
MN200876	Yunling	Taurine-Indicine	China
MN200873	Yunling	Taurine-Indicine	China
MN200872	Yunling	Taurine-Indicine	China
MN200871	Yunling	Taurine-Indicine	China
MN200870	Yunling	Taurine-Indicine	China
MN200869	Yunling	Taurine-Indicine	China
MN200868	Yunling	Taurine-Indicine	China
MN200867	Yunling	Taurine-Indicine	China
MN200866	Yunling	Taurine-Indicine	China
MN200865	Yunling	Taurine-Indicine	China
MN200864	Yunling	Taurine-Indicine	China
MN200863	Yunling	Taurine-Indicine	China
MN200862	Yunling	Taurine-Indicine	China
MN200861	Yunling	Taurine-Indicine	China
MN200860	Yunling	Taurine-Indicine	China
MN200859	Yunling	Taurine-Indicine	China
MN200858	Yunling	Taurine-Indicine	China
MN200857	Yunling	Taurine-Indicine	China
MN200856	Yunling	Taurine-Indicine	China
MN200855	Yunling	Taurine-Indicine	China
MN200854	Yunling	Taurine-Indicine	China
MN200853	Yunling	Taurine-Indicine	China
MN200852	Yunling	Taurine-Indicine	China
MN200851	Yunling	Taurine-Indicine	China
MN200850	Yunling	Taurine-Indicine	China

MN200849	Yunling	Taurine-Indicine	China
MN200848	Yunling	Taurine-Indicine	China
MN200847	Yunling	Taurine-Indicine	China
MN200846	Yunling	Taurine-Indicine	China
MN200845	Yunling	Taurine-Indicine	China
MN200844	Yunling	Taurine-Indicine	China
MN200843	Yunling	Taurine-Indicine	China
MN200842	Yunling	Taurine-Indicine	China
MN200841	Yunling	Taurine-Indicine	China
MN200840	Yunling	Taurine-Indicine	China
MN200839	Yunling	Taurine-Indicine	China
MN200837	Yunling	Taurine-Indicine	China
MN200835	Yunling	Taurine-Indicine	China
MN200822	Yunling	Taurine-Indicine	China
MN200816	Yunling	Taurine-Indicine	China
MN200811	Yunling	Taurine-Indicine	China
MN200808	Yunling	Taurine-Indicine	China
MN200807	Yunling	Taurine-Indicine	China
MN200806	Yunling	Taurine-Indicine	China
MN200805	Yunling	Taurine-Indicine	China
MN200804	Yunling	Taurine-Indicine	China
MN200803	Yunling	Taurine-Indicine	China
MN200802	Yunling	Taurine-Indicine	China
MN200801	Yunling	Taurine-Indicine	China
MN200800	Yunling	Taurine-Indicine	China
MN200799	Yunling	Taurine-Indicine	China
MN200798	Yunling	Taurine-Indicine	China
MN200797	Yunling	Taurine-Indicine	China
MN200796	Yunling	Taurine-Indicine	China
MN200795	Yunling	Taurine-Indicine	China
MN200794	Yunling	Taurine-Indicine	China
MN200793	Yunling	Taurine-Indicine	China
MN200792	Yunling	Taurine-Indicine	China
MN200790	Yunling	Taurine-Indicine	China
MN200789	Yunling	Taurine-Indicine	China
MN200788	Yunling	Taurine-Indicine	China
MN200787	Yunling	Taurine-Indicine	China
MN200784	Yunling	Taurine-Indicine	China
MN200783	Yunling	Taurine-Indicine	China
MN200782	Yunling	Taurine-Indicine	China
MN200781	Yunling	Taurine-Indicine	China
MN200780	Yunling	Taurine-Indicine	China
MN200779	Yunling	Taurine-Indicine	China

Table S2. Breeds and haplogroups

Breed	Subspecies	Common ancestral node	Age (X 1000 years)*
Creole	T1	T1	8.3
JBlack	T3	T3	8.4
Maremmana	T1, T2 & T3	T	16.9
Cinisara	T1&T3	T	17.9
Holstein-Friesian	T3 & T4	T	20.3
Nguni	T1	T	20.5
Marchigiana	T1	T	22.2
Korean	T2, T3 & T4	T	22.8
Angus	NA	NA	26.7
Cabannina	T2	T	31.5
Menofi	T1&T3	T	31.8
Chianina	T1, T3 & Q	QT	51.2
Domiaty	T1, T3 & Q	QT	52.6
Romagnola	T, Q & R	REPQT	161.2
Brahman	T1, T3, I1 & I2	REPQTI	314.5
Yunling	T1-T6, I1 & I2	REPQTI	325.4
Iraqi	I1, I2, T2 & T3	REPQTI	340.0
Iranian	I2 & T2	REPQTI	343.4

* - Estimated in this study and largely similar to previous estimates (Achilli et al. 2008; Achilli et al. 2009)
